# Population Attributable Risk of Unintentional Childhood Poisoning in Karachi Pakistan

**DOI:** 10.1371/journal.pone.0026881

**Published:** 2011-10-26

**Authors:** Bilal Ahmed, Zafar Fatmi, Amna R. Siddiqui

**Affiliations:** 1 Department of Medicine, Aga Khan University, Karachi, Pakistan; 2 Department of Community Health Sciences, Aga Khan University, Karachi, Pakistan; 3 King Saud University, College of Medicine Kingdom of Saudi Arabia, Riyadh, Saudi Arabia; Aga Khan University, Pakistan

## Abstract

**Background:**

The percentage of unintentional childhood poisoning cases in a given population attributable to specific risk factors (i.e., the population attributable risk) which can be calculated; determination of such risk factors associated with potentially modifiable risk factors, are necessary to focus on the prevention strategies.

**Methods:**

We calculated PARs, using 120 cases with unintentional poisoning and 360 controls in a hospital based matched case- control study. The risk factors were accessibility to hazardous chemicals and medicines due to unsafe storage, child behavior reported as hyperactive, storage of kerosene and petroleum in soft drink bottles, low socioeconomic class, less education of the mother and the history of previous poisoning.

**Results:**

The following attributed risks were observed: 12% (95% confidence interval [CI] = 8%–16%) for both chemicals and medicines stored unsafe, 19% (15%–23%) for child reported as hyperactive, 40% (38%–42%) for storage of kerosene and petroleum in soft drink bottles, 48% (42%–54%) for low socioeconomic status, 38% (32%–42%) for no formal mothers education and 5.8% (2%–10%) for history of previous poisoning. 48% of cases for overall study population which could be attributed to at least one of the six risk factors. Among girls, this proportion was 23% and 43% among boys. About half of the unintentional childhood poisoning cases in this Pakistani population could be avoided.

**Conclusion:**

Exposure to potentially modifiable risk indicators explained about half of the cases of unintentional poisoning among children under five years of age in this Pakistani population, indicating the theoretical scope for prevention of the disease.

## Introduction

Globally, every year one million deaths occur among children due to injuries [1]. Out of these, poisoning is the fourth leading cause after road traffic accident, burns and drowning[2]. Worldwide, children under five years of age account for about 15% of unintentional poisoning related deaths [3]. Children under five years of age contribute to about 23% of DALYs lost globally to poisoning [3]. The overall incidence of unintentional poisoning for United States is approximately 429.4/1000 children [4]. Low-middle income countries (LMIC) have relatively higher mortality rates for unintentional poisoning among children under five years of age [2], [5]. For low and middle income countries in EMRO region the mortality rates are 1.6 per 100,000 children, whereas for south Asian region it is 1.7 per 100,000 children [6]. A population based study that analyzed national health survey of Pakistan estimated 4.3% unintentional poising among children under five years of age [7].

The poisoning incidents are reported more among boys than girls [8]. It has been estimated that 30% young children who experience one such episode will have at least one further such incident before the age of 6 years [9].

Kerosene, medicines, household chemical, insecticides are the major sources of poisoning among children less than 5 years [10]–[12]. Unsafe storage of medicines and household chemicals, low parental education, low socioeconomic status, larger family size ≥4 children and history of previous poisoning are the key factors reported earlier [8], [13]–[16].

While various risk factors have been associated with unintentional poisoning among young children, the relationships are becoming increasingly relevant to health care policy and decision making in designing strategies for its prevention.

In this paper we present estimates for population attributable risk (PAR) for selected risk factors of unintentional poisoning reported at the tertiary care centers of Karachi, Pakistan. These PARs are the fraction of the total disease in our population that would not have occurred if the effect associated with the risk factor of interest were absent.

To our knowledge, this analysis constitutes the first attempt at a comprehensive population attributable risk study of the risk factors for unintentional childhood poisoning.

## Methods

Methods related to selection and enrolment of study participants have been described in detail earlier [16]. Briefly, 120 children under 5 years of age, with oral ingestion of any noxious substance were recruited from emergency room of three large tertiary care hospitals, after a definite diagnosis made by the attending physician. Children admitted for food poisoning, adverse drug reactions, and poisoning with animal venom were excluded. Three controls for every case were selected from the same hospitals ER within 48 hours of case identification, presenting with complaints other than poisoning. Controls were individually matched to cases for sex and age (+/−6 months) as these are the known confounders. To avoid a no response situation, children with symptoms of chronic illness such as known cases of cardiac disease, renal failure, chronic pulmonary disease, cancer patients, and road traffic accidents were excluded.

Care givers defined as parents/guardians were interviewed using a structured questionnaire, by trained medical students in local Urdu language. Information on socio-demographic characteristics of children and caregivers and storage practices of medicines and chemicals in the household of children were obtained. Economic status of a child's family/parents was determined using family income, household structure, and ownership. Information about parental education on the basis of number of schooling years completed, family type as nuclear or extended were also obtained. Respondents were asked about child behavior either hyperactive or not.

At the end of the interview, care givers were given health's educational material for information on proper storage, prevention and contact of poison control centers.

The risk factors under study were storage practices ascertained by accessibility to hazardous chemicals and medicines (both chemicals and medicines stored safe, either chemical or medicines stored safe, both chemicals and medicine stored unsafe); child behavior was reported as hyperactive; having a history of previous poisoning; storage of kerosene and petroleum in soft drink bottles, socioeconomic status of child's family determined by using family income, household structure, and ownership. Later, wealth index based on proportionate weighted sum of house hold assets was created [17], [18]. Mother education was defined as ≥10 yrs of education, <10 years of education, no formal education. The models with either accessibility to hazardous chemicals and medicines, storage of kerosene and petroleum in soft drink bottles, having a history of previous poisoning included the other two variables to control for reciprocal confounding.

The PAR is defined as the proportion of cases that can be related to a given risk factor (or set of risk factors) and is useful in assessing its impact at the population level. Estimation of population attributable risk (PAR) and corresponding 95% confidence intervals were obtained by using an approach based on conditional logistic regression [19], [20]. The following formula was used for the calculation of PAR%.



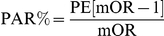



PE = probability of exposure of disease

mOR = matched odds ratios

By combining adjusted odds ratio estimates and the observed prevalence of the risk factors under study in the cases, this approach yields adjusted PAR estimates. The same logistic models were used for the odds ratio and PAR estimation, thereby allowing adjustment of PAR estimates for the same factors and in the same manner as for odds ratio estimates. For some risk factors (namely, accessibility to hazardous chemicals and medicines, socioeconomic status and mother's education level), level specific PARs were estimated in addition to overall PARs. Overall PARs measure the impact of any exposure to the risk factor, while level specific PARs measure the impact of a specific level of exposure. PARs were estimated for combination of risk factors as well as for each separate risk factor. It should be noted that because the logistic model assumes a multiplicative effect on the odds ratio scale, the PAR for the combination of two or more risk factors is usually less than the sum of the PARs for each risk factor.

Causality is critical while interpreting PAR for a disease to be caused by a risk factor and purging from the population could avert further incidences. However, regardless of proven causality, PARs are generally estimated for well established risk factors. For more approximate risk factors, PAR estimates can be regarded as measuring their potential impact on disease incidences and the potential reduction in disease incidence that could be attained for their elimination were that they later proved to be causal [21]. We present PAR estimates for both type of risk factors (established and non established causality) in the results and clearly distinguish between them.

The study was approved by ethical review committee of Aga Khan University. Written informed consent was obtained from participant's parents or guardians.

## Results

Cases and controls were similar in distribution by age (p-value 0.98) and gender (p-value 0.95) shown in table 1. For the main risk factors of unintentional childhood poisoning under study, PAR along with 95% CI for all children as well as stratified on gender are presented in table 2. Estimates of matched odds ratios that influence the PAR are also shown.

**Table 1:** Characteristics of 120 unintentionally poisoned children and 360 matched controls in tertiary care public and private hospitals in Karachi.

Variable	Case		Controls	
**Age (months)**				
≤12 months	13		39	
>12–≤24 months	38		116	
>24–≤36	34		109	
>36–≤48	25		67	
>48–≤60	10		29	
**Accessibility to hazardous chemicals and medicines**	n	%	n	%
Both chemicals and medicines stored safe	61	(50.8)	238	(66.1)
Either chemical or medicines stored safe	42	(35)	101	(28.1)
Both chemicals and medicine stored unsafe	17	(14.2)	21	(5.8)
**Child reported as hyperactive**				
Yes	94	(78.3)	136	(37.8)
No	26	(21.7)	224	(62.2)
**Storage of kerosene and petroleum in soft drink bottles**				
No	55	(45.8)	68	(18.9)
Yes	65	(54.2)	292	(81.8)
**Socioeconomic status**				
Upper	13	(10.8)	81	(22.6)
Middle	42	(35)	149	(41.5)
Low	65	(54.2)	129	(35.9)
**Mother's education level**				
≥10 yrs of education	26	(21.7)	134	(37.2)
<10 years of education	33	(27.5)	107	(29.7)
No formal education	61	(50.8)	119	(33.1)
**History of previous poisoning**				
No	112	(93.3)	355	(9.6)
Yes	8	(6.7)	5	(1.4)

**Table 2:** Matched Odds ratios (mORs), population attributable risks (PARs), and 95% confidence intervals (CIs) for the main risk factors for unintentional childhood poisoning in a matched case control study in Karachi.

Risk Factors	mOR	95% CI	All Children		Boys		Girls	
			PAR	95% CI	PAR	95% CI	PAR	95% CI
**Accessibility to hazardous chemicals and medicines**								
Both chemicals and medicines stored safe	1							
Either chemical or medicines stored safe	1.5	0.8–2.8	11.6	0.7 to 15	11	7 to 15	12.4	8.5 to 16.3
Both chemicals and medicine stored unsafe	5.6	1.9–16.7	12	8 to 16	12	8 to 16	11.2	7 to 15
**Child reported as hyperactive**								
No	1							
Yes	8.2	4.6–16.1	19.1	15.1 to 23	72	68 to 76	63	59 to 67
**Storage of kerosene and petroleum in soft drink bottles**								
No	1							
Yes	3.8	2.0–7.3	40	38 to 42	43	39 to 46	36	32 to 40
**Socioeconomic status**								
Upper	1							
Middle	2.5	0.9–6.6	21	15.1 to 26.8	18.2	14 to 22	25	21 to 28
Low	9.2	2.8–30.1	48	42.1 to 53.8	48	44 to 51	49	45 to 53
**Mother's education level**								
≥10 yrs of education	1							
<10 years of education	2.2	0.9–5.2	15	9.1 to 20.8	16	12 to 19.7	16	12 to 19
No formal education	4.2	1.8–9.6	38	32.1 to 42.6	37	33 to 41	40	0.40 to 44
**History of previous poisoning**								
No	1							
Yes	8.6	1.7–43.5	6	2 to 10	6.3	2.4 to 10	5.2	1.2 to 9.1

Analysis showed that odds of unsafe storage of either chemical and medicines were 50% more in cases than controls, yielding a PAR of 11%. However, for both chemicals and medicine stored unsafe PAR of 12% is about same for both boys and girls.

Child behavior reported as hyperactive, were 8.2 times in cases as compared to controls giving a PAR of 19. Boys also had a relatively higher prevalence of hyper-activeness so that its impact was much pronounced among boys (PAR = 72) compared to girls (PAR = 63). Another modifiable risk factor, storage of kerosene and petroleum in soft drink bottles is significantly 3.8 times more among cases then controls resulted in a PAR of 40%. The effect of this improper storage is more marked among boys (PAR = 43) than girls (PAR = 36). Socioeconomic status showed a trend of increasing PAR with decreasing economic status. Belonging to low socioeconomic status is significantly associated with unintentional poisoning with odds 9.2 times among cases than controls. This resulted in the largest PAR of 48% with gender not making a difference. Mother's education level of <10 years gave an overall PAR of 15% whereas for mother with no formal education, the PAR was 38%. History of previous poisoning was a major contributor to unintentional poisoning among this population, with a PAR of 5.8%. These PAR were slightly larger for boys (PAR = 6.3) than girls (PAR = 5.2).

In the overall study population, 48% of cases could be attributed to at least one of the six well established risk factors. Among girls, this proportion was 23% and it rose to 43% among boys.

## Discussion

This study indicates that about half of the unintentional childhood poisoning cases in this Pakistani population could be avoided by the intervention on a few selected and modifiable risk factors: safe storage of both chemicals and medicines, avoid storing kerosene and petroleum in soft drink bottles, improving mother's educational status and to some extent by reducing hyper- activeness among children. The point estimates of these PARs are somewhat larger for boys as compared to girls.

These findings have considerable public health relevance, since they suggest that it is possible, at least in principle to prevent an appreciable proportion of unintentional childhood poisoning cases by changing a few selected household environmental factors identified previously [14], [16]. Majority of unintentional poisoning occurs insides home, therefore a holistic approach which targets household environment would help in curtailing the burden [22].

Unsafe storage of household chemicals and medicines are 5.6 times more reported among cases than controls and we can prevent about 12% of such incidents, a finding consistent with a study reported from Brazil [23]. In addition to unsafe storage, availability of household chemicals and medicines in non-child resistant containers make these age group children more prone for such incidents. Kendrick et al indicated that home safety education and provision of safety equipments increases safe storage of medicines and cleaning products [24].

Childhood behavior as hyperactive is another important determinant identified by our study, a finding consistent with earlier studies [15], [25]–[27]. Furthermore, it is also observed that the behavior of hyperactivity makes children prone to the ingestion of harmful substances. However, such personality characteristics of children when accompanied with poor storage practices at home resulted in a greater risk of ingestion of hazardous substances. Our PARs for hyper-activeness among boys should be interpreted with caution until these associations are confirmed in future studies. These PAR estimates suggest that, if a causal link is established, hyper activeness may be an important determinant of unintentional childhood poisoning.

Another potentially modifiable factor, storage of kerosene in soft drink bottle is not uncommon in developing countries [28], [29]. These containers are often kept in easily accessible places such as the kitchen floor, a low table or a low shelf without a resistant closure. Children often mistake kerosene oil for bottled soft drink due to its color. This has long been analyzed, and the results of this study are consistent in terms of OR. This study adds PAR of 40% attributed to this factor adds further substantiation to the existing literature.

Low SES yields highest Odds Ratio (mOR = 9.2, 95% CI 2.8–30.1) a findings consistent with studies from developing as well as developed countries [7], [8], [30], the PAR reached 48%. This SES gradient is not only associated with exposure to poisonous substances but is also related with several other factors such as poor storage, literacy of parents and household fuel consumption as the use of kerosene is common in low socioeconomic group [31], [32]. However, in our data SES does not have any correlation with storage practices (p-value >0.05); moreover gender does not play a significant role with SES gradient.

Another consistent cause is the history of previous poisoning incidence, as this increase the likelihood of later occurrence [9], [13], [33]. The elimination of this factor by providing safer household environment along with extensive counseling to parents would help in preventing of 6% of poisoning in children.

Nonetheless, though statistically insignificant, the protective association between ≤2 sibling's and extended family type with childhood poisoning indicates this to be a good indicator for the prevention of such injuries [16].

Other risk factors for unintentional poisoning have been suggested, such as marital status of parents as living apart, number of siblings' ≥3, medicine users at home [14]. We have not presented results on these variables in the present paper because the epidemiologic evidence to date is inconclusive or because no clear relation was found in our study population.

The main strengths of this study are well powered sample size and its multi center design. Matching was performed on age and gender as these are the known confounders. The estimation of PARs is ideal for population based studies, however in our part of the world we see more serious poisoning due to hydrocarbons, pesticides and cleaning agents, hence the chances of picking subjects from the emergency rooms of tertiary care settings are high. We conducted our study at two large public and private care centre's. Nevertheless, the possibility of missing less severe poisoning cannot be ignored. Regrettably, there are no published surveillance reports or data from poison control centers to infer our results. Although, the under reporting of cases who do not seek the treatment at health care facility remains with the surveillance data as well. We cannot overlook the possibility of differential recall for parents reporting previous poisoning events as injury data does not support more than one year of reliable recall. However, in our setting we see more serious poisoning which inevitably is a hard outcome, making, it unlikely that recall of these events would have compromised in many important ways. Moreover, the study population comprised children less than five years of age hence, the period of recall is limited to five years in extreme cases where most parents assume to have a reliable recall for their children, as shown in previous study as well [13], [34]. Finally, our PAR estimates apply to the city of Karachi, and extrapolations to other areas may be valid only for populations with a similar mix of exposures and susceptibility factors.

Despite the limitations outlined above, we present a robust methodology for calculating quantitative epidemiological measures of disease burden which provides policy makers and health service administrators with an important tool to prioritize health services and prevention strategies.

In summary, our results show that about 48% of cases of unintentional childhood poisoning in Karachi are associated with unsafe storage of chemicals and medicines, storage of kerosene and petroleum in soft drink bottles, low mother education and history of previous poisoning. These poisoning cases can be prevented by various interventions particularly safe storage and legislative policy for the child resistant packaging. The findings also indicate that at least 52% of cases remain unexplained which highlights the need for further research into other household environmental and behavioral determinants of this undoubtedly preventable disease.
